# The Effectiveness of Transcranial Direct Current Stimulation (tDCS) in Binge Eating Disorder (BED)—Review and Insight into the Mechanisms of Action

**DOI:** 10.3390/nu16101521

**Published:** 2024-05-17

**Authors:** James Chmiel, Donata Kurpas, Filip Rybakowski, Jerzy Leszek

**Affiliations:** 1 Institute of Neurofeedback and tDCS Poland, 70-393 Szczecin, Poland; 2 Department of Family and Pediatric Nursing, Faculty of Health Sciences, Wrocław Medical University, 51-618 Wrocław, Poland; 3 Department and Clinic of Psychiatry, Poznan University of Medical Sciences, 61-701 Poznań, Poland; 4 Department and Clinic of Psychiatry, Wrocław Medical University, 54-235 Wrocław, Poland

**Keywords:** tDCS, transcranial direct current stimulation, binge eating disorder, non-invasive brain stimulation, neurostimulation, neuromodulation, eating disorder

## Abstract

Introduction: Binge eating disorder (BED) is the most common eating disorder among those contributing to the development of obesity, and thus acts as a significant burden on the lives and health of patients. It is characterized by complex neurobiology, which includes changes in brain activity and neurotransmitter secretion. Existing treatments are moderately effective, and so the search for new therapies that are effective and safe is ongoing. Aim and Methods: This review examines the use of transcranial direct current stimulation (tDCS) in the treatment of binge eating disorder. Searches were conducted on the PubMed/Medline, Research Gate, and Cochrane databases. Results: Six studies were found that matched the review topic. All of them used the anodal stimulation of the right dorsolateral prefrontal cortex (DLPFC) in BED patients. tDCS proved effective in reducing food cravings, the desire to binge eat, the number of binging episodes, and food intake. It also improved the outcomes of inhibitory control and the treatment of eating disorder psychopathology. The potential mechanisms of action of tDCS in BED are explained, limitations in current research are outlined, and recommendations for future research are provided. Conclusions: Preliminary evidence suggests that the anodal application of tDCS to the right DLPFC reduces the symptoms of BED. However, caution should be exercised in the broader use of tDCS in this context due to the small number of studies performed and the small number of patients included. Future studies should incorporate neuroimaging and neurophysiological measurements to elucidate the potential mechanisms of action of tDCS in BED.

## 1. Introduction

Binge eating disorder (BED) is the most common eating disorder (ED) and affects 3–5% of the US population [1]. It is included as a diagnostic entity in the Fifth Edition of the Diagnostic and Statistical Manual of Mental Disorders (DSM-5) [2,3]. It is characterized by recurrent (≥1 per week for 3 months), short (≤2 h) episodes of binge eating [4]. The affected person loses control and consumes a significant amount of food, eating far more than what he or she would eat under normal circumstances and times. During a binge eating episode, patients may eat large amounts of food. Even though they do not feel physically hungry or have metabolic needs [5], they eat faster than usual and until they feel the discomfort of over-satiation [6]. Binge eating episodes are associated with guilt and anxiety. Patients with BED often eat secretly, being ashamed of their behavior and lack of ability to control food intake [7].

A diagnosis of BED can be conducted when there are uncontrollable eating episodes lasting for 2 h, for at least 2 days a week for 6 months. At the same time, patients do not engage in compensatory behaviors typical of bulimia, such as inducing vomiting, using laxatives, and intense physical activity [8]. In the International Classification of Diseases and Related Health Problems, Tenth Edition (ICD-10), BED has been classified among Other Eating Disorders (F50.81) without any further diagnostic criteria [2]. New guidelines have been added in ICD-11 that allow binge eating and eating control to be assessed subjectively and objectively. 

BED is the most common eating disorder, but its prevalence varies due to the different definitions of compulsive eating [8]. It is more common in women (3.5%) than in men (2%) [4,9]. The lifetime prevalence in young individuals is >1% [10]. A meta-analysis by Qian et al. [11] estimated the lifetime prevalence of BED at 2.22%. It is common in obese people (5% to 30%) [12,13], but a significant proportion of people with BED (17–30%) are of normal weight [14].

The coexistence of other psychiatric conditions often characterizes patients with BEDs. People with BED have a distorted image of their body and its weight [8] and worry more about their shape than people who do not have BED [15,16]. According to Grilo [17], 67% of patients with BED have at least one additional mental disorder during their lifetime. They are more likely to suffer from depression [8], anxiety [18], substance-use disorders [19], and other mental disorders [20]. A meta-analysis by Friborg et al. [21] of nine studies estimated the prevalence of personality disorders in people with BED to stand at 29%. The most common were avoidant personality disorder (12%) and obsessive–compulsive and borderline disorders (10%). People with BED are more likely to seek treatment for mood and anxiety disorders than for BED [22]. Undiagnosed BED means that the treatment of mental disorders may be suboptimal, as prescribed antidepressants often increase appetite, thus worsening inappropriate eating behavior [23]. People with BED have sleep disorders [24], heightened risk of metabolic syndrome [25] and fibromyalgia [20], an increased incidence of irritable bowel syndrome, earlier menarche, neck–shoulder pain, lower back pain, chronic muscular pain, impairment due to physical health [24], and attention deficit hyperactivity disorder (ADHD) [10]. Obesity is a common disease coexisting with BED and both diseases increase the risk of dyslipidemia, diabetes, and hypertension [25]. Further data suggest that the functioning of the reproductive and cardiovascular systems may also be impaired in people with BED [26]. In terms of risk factors, alcohol abuse, the worsening of depressive symptoms, taking medication for psychiatric reasons, and low self-esteem all increase the likelihood of developing BED [27].

The treatment of BED is aimed at reducing the frequency of binge eating and eating-related cognitive disorders, lowering BMI and improving metabolic health (in patients with obesity), and improving mental health (in patients with mental disorders) [4]. The methods used for this include cognitive behavioral therapy (CBT) [28], pharmacological therapy [29], or a combination of both methods [4]. The APA recommends a combined approach of CBT and treatment with selective serotonin reuptake inhibitors [4].

## 2. Neurobiology of BED

The neurobiology of BED is similar to that seen in substance abuse disorders [5]. Like drugs, food activates brain reward pathways [30,31]. Food cues induce the same neural plasticity and gene expression in the mesolimbic–cortical reward pathway and in the brain regions responsible for learning and memory [5,32]. Research on the neurotransmitters involved in BED has focused on neurotransmission in the endogenous opioid and dopaminergic systems because of their functions in motivation, cognitive control [33], and the rewarding of eating behavior [34]. Endogenous opioids in the nucleus accumbens have been linked to the hedonic properties of food and are dysregulated in individuals with BED [35]. Eating sweet foods increases opioid receptor binding in the reward system [5,36]. Opioid receptor binding in the insular cortex is reduced in people with BED [37]. In a rat study, the intake of sweet liquids was associated with increased mu-1 opioid receptor binding in the hippocampus, cingulate cortex, locus coeruleus, and accumbens shell [38].

Regarding dopamine, eating disorders have been linked to dopaminergic dysregulation in the central nervous system [39,40]. Eating tasty food activates dopaminergic neurons in the nucleus accumbens (NAc), ventral striatum, and other reward centers [32]. The development of BED has been linked to repeated stimulation of this system to alleviate the psychological effects of stress [41]. Further evidence of BED-induced changes in dopaminergic pathways supports the fact that binge eating is addictive. In a rat study, binge eating increased dopamine binding in the nucleus accumbens and striatum [42]. In another study, rats fed on palatable food showed decreased dopamine D2 binding in the striatum [39]. In the Colantuoni et al. [38] study, rats addicted to drinking sugary solutions showed increased dopamine D1 receptor binding in the NAc and decreased D2 receptor binding in the dorsal striatum. Overall, palatable food consumption likely increases reward sensitivity and obesity, and decreases dopamine release. However, the development of behaviors leading to food addiction may be mediated by an imbalance associated with reduced dopamine D2 receptor levels coupled with relatively higher levels of dopamine D1 receptor signaling in the dorsolateral striatum [34]. Detailed information on the neurobiology of BED is included in [5,6,34].

As shown above, BED is associated with many changes in the brain, which primarily affect the reward systems. Current treatments are moderately effective and interventions targeting the brain directly are needed. One of them may be transcranial direct current stimulation (tDCS), a non-invasive technique that has already been proven to work in terms of reducing food cravings and drug cravings. 

## 3. Transcranial Direct Current Stimulation (tDCS)

tDCS involves the application of low-intensity electrical currents to the scalp in roder to modulate brain activity [43]. The technique has gained increasing attention in recent years due to its potential therapeutic applications in various neurological and psychiatric disorders and its potential to enhance cognitive function in healthy individuals. During tDCS, two or more electrodes are placed onto the scalp, and a low-intensity direct current is applied to modulate the activity of underlying neural circuits [44]. The anode is typically placed over the brain region intended for stimulation, while the cathode is placed onto a remote area. The current flow between the electrodes leads to changes in the membrane potential of neurons in the underlying brain tissue, which can modulate their firing rate and synaptic plasticity [45]. The effects of tDCS on brain activity are thought to be mediated by changes in the excitability of cortical neurons, synaptic plasticity, and neuroplasticity. The exact mechanisms of action are still the subject of ongoing research, but it is thought that tDCS may enhance or suppress the activity of specific neural networks depending on the location and polarity of the electrodes.

Several reviews have investigated the effectiveness of tDCS in EDs, including BED, with promising results [46,47,48,49,50]. However, these investigations have included all EDs, which may lead to misleading conclusions about the effectiveness of BED alone. In addition, there have recently been new clinical trials in BED that have not been included. Therefore, the purpose of this review is to analyze all studies investigating the effectiveness of tDCS in the treatment of BED, to examine the effects of stimulation on various aspects of BED and cognitive constructs, to try to elucidate potential mechanisms, to propose new therapy targets, and to investigate safety. Moreover, a review of research on the use of tDCS in BED may be useful because, as previously mentioned, the neurobiology of BED is similar to that of substance addiction. Several reviews have shown that tDCS is effective in treating nicotine [51] and alcohol use disorder [52]. It is therefore worth comparing the effectiveness of tDCS between substance addiction and BED.

## 4. Methods

### 4.1. Data Sources and Search Strategy

For this review, J. Ch., D. K., F. R., and J. L. performed an independent online search using predefined criteria. The following combined keywords were used: “transcranial direct current stimulation” OR “tDCS” AND “binge eating disorder” OR “BED”. We considered publications in the PubMed/Medline, Research Gate, and Cochrane databases, with an access date of March 2024 and publication dates ranging from January 2000 to March 2024.

### 4.2. Study Selection Criteria

Eligibility criteria included clinical trials conducted in English that were published from 2008 to 2024. We considered studies investigating the effects of tDCS on binge eating disorder. The exclusion criteria encompassed articles that still needed to be published in English and reviews.

### 4.3. Screening Process

Multiple screening processes were implemented to guarantee the inclusion of pertinent research and the rejection of those that did not satisfy the predetermined criteria. Independent reviewers J. Ch., D. K., F. R., and J. L. thoroughly examined the titles and abstracts during the first screening process.

#### 4.3.1. Title and Abstract Screening

To find studies that would fit the inclusion requirements, each reviewer evaluated the titles and abstracts of the records they could find independently. The relevance to transcranial direct current stimulation and its impact on binge eating disorder were the main screening criteria used during this phase.

#### 4.3.2. Full-Text Assessment

After screening for titles and abstracts, the chosen papers were subjected to a thorough full-text evaluation. To ascertain if the full articles satisfied the comprehensive eligibility requirements, reviewers focused on adding clinical trials to the study that were carried out in English and released between January 2000 and March 2024. 

## 5. Results

**Figure 1:**
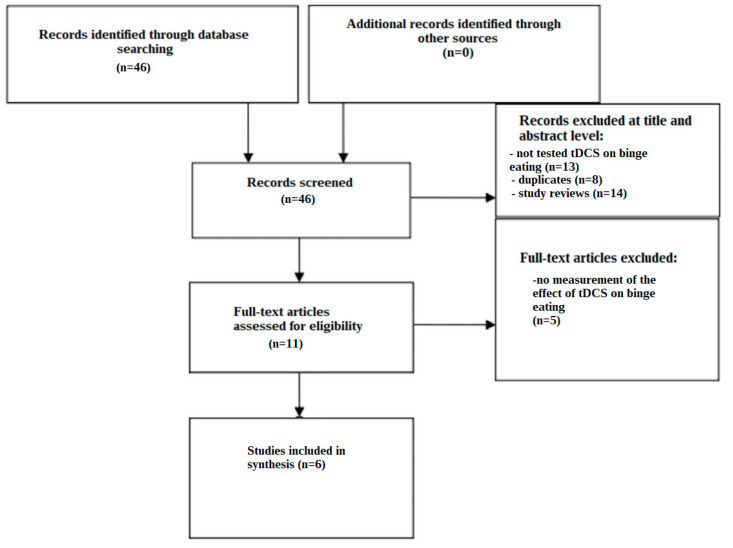
Flow chart depicting the different phases of the systematic review.

The screening process is illustrated in a flow chart (Figure 1). All told, 46 studies were found using the search techniques used in the databases. Based on the assessment of their titles and abstracts, 35 papers were eliminated. The reasons for this included the exclusion of study reviews (n = 14), the removal of duplicates (n = 8), and the lack of testing for tDCS in binge eating disorder (n = 13). A total of 11 papers were then found and subjected to a thorough full-text evaluation. Five of these studies were disqualified because the effect of tDCS on binge eating disorder was not measured. After thoroughly examining the texts, six articles were found to be suitable for inclusion.

The studies found were published between 2016 and 2023. A total of 165 patients were enrolled. Two studies were proofs of concept [53,54] and four studies were RCTs [55,56,57,58]. Random assignment occurred in five studies [54,55,56,57,58]. All studies used sham stimulation for the control group. In four studies [53,55,56,57], patients and experimenters were blinded; in the study by Burgess et al. [54], only patients were blinded. A current of 1 or 2 mA was used. As for the montage of the electrodes, a unipolar montage was the most common, in which one electrode was placed on the scalp and the other was placed extracranially on the left deltoid muscle [53,54,56,57,58]. In the one remaining study [55], both electrodes were placed over the brain and the same current was passed through the anode and cathode.

### 5.1. Summary of Included Studies

The studies included are summarized in Table 1. A crossover design, double-blind, randomized, and placebo-controlled proof-of-concept study by Max et al. [53] examined the impact of food-modified antisaccade tasks and the right dlPFC anodal tDCS on response inhibition in a BED-diagnosed sample. Participants diagnosed with BED included both normal-weight and obese adults. Sixteen subjects participated in the 1 mA condition, and 15 subjects participated in the 2 mA condition. Participants received either real tDCS or sham tDCS, which were given in a random order. tDCS was delivered for 20 min. Using the international 10–20 electrode placement system, the cathode was positioned in an extracephalic manner on the left deltoid muscle and the anode was positioned above F4 (right DLPFC). Response inhibition was measured by an antisaccade task, which required the suppression of dominant responses (i.e., saccade) towards a newly appearing picture in the visual field. The self-reported frequency of binge eating episodes was used to measure binge eating frequency. 

In a proof-of-concept study by Burgess et al. [54], 30 adults with BED or subthreshold BED and obesity participated. Participants received one session of real or sham tDCS (20 min, 2 mA). The cathode was positioned over the left DLPFC (F3), and the anode was positioned over the right DLPFC (F4). The outcome measures used were Binge Eating Scale (BES), employed to measure binge eating frequency; the Dutch Eating Behavior Questionnaire-Restraint Subscale (DEBQ-R), used to measure food intake; and the Palatable Eating Motives Scale (PEMS), used to measure food craving and desire to binge eat.

The aim of the randomized controlled trial of Gordon et al. [55] was to explore participants’ experience of approach bias modification training (ABM) with tDCS for BED. ABM is a cognitive intervention method that aims to mitigate automatic cognitive bias towards substance use or unhealthy behaviors by retraining the brain’s automatic response tendencies. Fifteen participants received six sessions of concurrent ABM training with either real (n = 6) or sham (n = 9) tDCS. The current intensity was 2 mA, the anode was applied to the right DLPFC (F4), and the cathode was applied to the left DLPFC (F3). The duration of the intervention was not reported, and the outcome measurements needed to be provided. The primary outcome was the frequency of binge eating.

The study of Giel et al. [56] sought to show the clinical use and viability of a tDCS-enhanced inhibitory control training program in efforts to lower BE episodes, performing a monocentric clinical phase II double-blind randomized trial with two parallel arms. Six sessions of food-related inhibitory control training were randomly paired with 2 mA real or sham tDCS of the right DLPFC for forty-one adult outpatients with full-syndrome BED. No reference electrode data were used. The tDCS session lasted for 20 min. The primary outcome was the difference in BE frequency between the baseline values and those from four weeks after treatment termination (T8; primary) and twelve weeks later (T9; secondary). The binge eating frequency was measured by the Eating Disorder Examination (EDE). To assess inhibitory control capacity, we measured the course of the error rate (%) of the food-related eye-tracking task from the beginning of the training (T1) over each training session until immediately after the training (T7) as compared to the baseline values (T0). Feelings of hunger were measured by the Three-Factor Eating Questionnaire (TFEQ). 

In the study of Beaumont et al. [57], two sessions of double-blind, randomized, counterbalanced anodal, and sham tDCS were completed over the right dorsolateral prefrontal cortex at a frequency of 2 mA for 20 min by seventeen females with mild-to-moderate binge eating behavior. Prior to the study, the participants’ weights were consistent for three months, with most of them (n = 9) being healthy weights; two were classed as obese, while six were overweight. The cathode was placed over the occipital zero point (Oz). Pre- and post-tDCS measures included the subjective appetite visual analogue scale (VAS), which measured hunger; the Food Craving Questionnaire-State (FCQ-S), measuring food craving; the Leeds Food Preference Questionnaire (LFPQ), measuring subjective desire or craving for foods; and Control of Eating Questionnaire (CoEQ), measuring craving.

A computer-based inhibitory control training program augmented by tDCS was examined in a randomized controlled experiment by Max et al. [58] at a 6-week follow-up. Patients were assessed in terms of eating behavior, general impulsivity, food cravings, and eating disorder psychopathology in both evaluations using an experimental virtual reality paradigm. Some 31 participants took part in the study (active tDCS n = 15, sham tDCS n = 16). The anode was placed on the right DLPFC (F4), and the cathode was positioned extracephalic on the left deltoid muscle. We carried out 6 sessions at an intensity of 2 mA. tDCS sessions lasted for 20 min. The Food Craving Questionnaire-State (FCQ-S) was used to measure craving. General eating disorder psychopathology and binge eating frequency were measured via Eating Disorder Examination (EDE).

### 5.2. Effects on Binge Eating Episodes and Frequency

In [53], a significant decrease in self-reported binge eating episodes over time was observed, but only in the group that had 2 mA stimulation, and there was no change in the group that had 1 mA stimulation.

In [54], a Wilcoxon signed-rank test indicated that there was no effect of tDCS on binge eating frequency, as measured by BES.

In [55], six participants observed a reduction in binge eating episodes (ABM and real tDCS n = 2; ABM and sham tDCS n = 4), ranging from ‘a little bit’ to a ‘huge difference’.

In [56], BE frequency in the real tDCS group was reduced from 18.6 to 4.4 (T8) and to 3.8 (T9).

In [58], the number of binge eating episodes decreased (from 17.35 to 6.26).

### 5.3. Effects on Inhibitory Control (Response Inhibition)

In [53], all patients improved over the three measurement points concerning the error rate and latencies of correct antisaccades. The stimulation did not affect error rates, but the group that had 2 mA stimulation improved with faster latencies of correct antisaccades compared to sham stimulation, and the group that had 1 mA stimulation showed slower latencies.

In [56], the results showed a significant improvement over the training period and showed a significant effect at T7 as compared to T0 in both study arms. 

### 5.4. Effects on Food Intake

In the study of Burgess et al. [54], tDCS significantly reduced food intake, as measured by DEBQ-R. Participants ate fewer total kcals in the lab after tDCS (614.50 ± 55.5) versus after sham (689.54 ± 60.8), with an 11% reduction. After tDCS, participants ate 324.72 ± 30.5 kcals of the preferred food versus 393.52 ± 36.3 kcals after sham, a 17.5% reduction.

### 5.5. Effects on Food Craving, Hunger and Desire to Binge Eat

In [53], tDCS significantly reduced food craving, as measured by PEMS. The decreased the intake of palatable food due to Reward Enhancement motives accounted for 20% of the variance in the reductions in food craving. Additionally, tDCS significantly reduced the desire to binge eat, which is also measured by PEMS. An r-ANOVA showed that tDCS reduced desire to binge eat on the day of the stimulation, roughly 5–6 h post-stimulation, in men only.

In [56], the results of the TFEQ showed that this measure decreased from 10.6 (T0) to 9.3 (T7 and T8) after real tDCS.

In [57], studying the appetite visual analogue scale (VAS), hunger levels following active tDCS treatment grew to meet those of post-sham stimulation, indicating a substantial change from pre-to post-tDCS (F(1, 15) = 6.796, *p* = 0.020, = 0.312, BF10 = 0.188). This effect was no longer significant (F(1, 30) = 0.610, *p* = 0.441, = 0.020, BF10 = 0.680) when baseline hunger was taken into account. When comparing measures of fullness (F(1, 15) = 1.282, *p* = 0.275, = 0.079, BF10 = 0.040), prospective consumption (F(1, 15) = 2.606, *p* = 0.127, = 0.148, BF10 = 0.063), and desire to eat (F(1, 15) = 1.452, *p* = 0.247, = 0.088, BF10 = 0.054), no significant differences were observed between the active and sham tDCS. Bayes factors suggested that there was moderate-to-strong evidence in favor of the null hypothesis. Regarding LFPQ, all the assessments showed no significant impacts, except for explicit desire and a preference for HFSW foods. The preference for HFSW foods rose after active tDCS and fell after sham tDCS for both explicit like and desire. ANCOVA was used to assess if the difference in baseline hunger was the cause of these substantial effects; when hunger was considered, the difference between pre- and post-tDCS ceased to be significant. Regarding FCQ-S, scores for food cravings after active versus sham procedures did not differ. In the CoEQ results, savory food cravings were close to significant (active 45.3 ± 17.9 mm, sham 49.4 ± 20.6 mm) (t(13) = 2.128, *p* = 0.053), indicating that savory food cravings decrease when active procedures are followed.

In [58], the total FCQ score improved (from 38.26 to 37.97).

### 5.6. Effects on Eating Disorder Psychopathology

The EDE score improved in [58] (from 2.61 to 2.04).

## 6. Discussion

This review includes a wide range of research that uses tDCS to examine how it affects food intake, food intake episodes, inhibitory control, and related psychopathological features in people with binge eating disorder. Despite having different methods, the trials provide important information about the potential of tDCS as an adjuvant therapy strategy for BED.

### 6.1. Impact of tDCS on Binge Eating Episodes and Frequency

The diverse outcomes observed across the studies underscore the nuanced impact of tDCS on binge eating episodes. While Burgess et al. [54] found no significant effect on binge eating frequency, Max et al. [53] reported a marked reduction in self-reported episodes, particularly in the group receiving 2 mA stimulation. Gordon et al. [55] noted fewer binge eating episodes in individuals undergoing approach bias modification training with real tDCS. Contrarily, real tDCS led to significant reductions in binge eating frequency in the studies by Giel et al. [56] and Max et al. [58]. The discrepancy in findings may be attributed to the inclusion of individuals with subthreshold BED in the study by Burgess et al. [54], possibly diluting the impact on binge eating episodes. Overall, stimulation of the right DLPFC at 2 mA appears to be effective in decreasing the frequency of BED episodes, suggesting the potential utility of adjunct interventions, such as approach bias modification training, to augment the effects of tDCS synergistically.

### 6.2. Impact of tDCS on Inhibitory Control (Response Inhibition)

Across studies, improvements in inhibitory control, particularly in response inhibition tasks, were consistently observed. Max et al. [53] reported faster latencies of accurate antisaccades with 2 mA stimulation, indicative of enhanced inhibitory control. Similarly, Giel et al. [56] noted a significant improvement in both study arms during the training phase. These findings suggest that the stimulation of the right DLPFC at 2 mA may enhance inhibitory control in individuals with BED, with discernible effects after a single session.

### 6.3. Impact of tDCS on Food Intake

tDCS demonstrated a significant impact on food intake, as evidenced by decreased consumption following stimulation. Burgess et al. [54] reported a substantial decrease in overall food intake and calories from preferred meals after treatment with active tDCS compared to sham, with effects evident after a single session. Song et al.’s [59] meta-analysis suggests that a greater number of tDCS sessions may further enhance the reduction in food intake, warranting exploration in future studies in order to optimize treatment efficacy.

### 6.4. Impact of tDCS on Food Craving, Hunger, and Desire to Binge Eat

The evidence suggests that there are varying outcomes regarding the effects of tDCS on hunger, food cravings, and binge eating tendencies. Burgess et al. [54] reported a notable decrease in food cravings and binge eating inclination following tDCS, contrasting with the findings Giel et al. [56] in terms of reduced TFEQ scores post-real tDCS. Notably, Beaumont et al. [57] highlighted the moderating role of baseline hunger levels on the effects of active tDCS, indicating potential variability in individual responses. Moreover, individuals with elevated BMI and BED tend to exhibit heightened food craving [60,61,62], underlining the necessity for future studies to stratify participants based on similar BMI values and confirmed clinical BED to account for potential differences in the effects of tDCS across different BED severity levels. Sex-specific differences were evident, with men showing greater susceptibility to tDCS-induced reductions in food cravings and binge eating desires compared to women, as observed by Ray et al. [63]. Additionally, Ray et al. [64] highlighted the influence of expectations on hunger reduction and eating behavior, indicating that individuals who believed they received active tDCS experienced more significant improvements in hunger and eating compared to those who believed they received sham tDCS, regardless of the actual stimulation received. It is worth emphasizing that persons with elevated BMI and BED display higher food craving [60,61,62]. Therefore, will be necessary in future studies to create groups with similar BMI values and with confirmed clinical BED, because the impact of tDCS on people with different degrees of BED (subBED or no BED) and on people with different BMI may be different. Men were more susceptible to the effects of tDCS on food cravings and the desire to binge eat than women, suggesting that the effect on craving reduction may be sex-specific. Ray et al. [63] also reported on the impacts of sex, showing that only women with low attentional impulsivity saw a decrease in food cravings. Expectations also influence the impact of tDCS on reducing hunger and eating. In the study of Ray et al. [64] into obese people, it was shown that people who claimed (regardless of the actual type of stimulation) that they had participated in active tDCS achieved better results in terms of reducing hunger and eating than people who claimed that they were in the sham tDCS group.

### 6.5. Impact of tDCS on Eating Disorder Psychopathology

tDCS exhibited promising effects on the psychopathology of eating disorders, as reflected by the improvements in Eating Disorder Examination scores post-stimulation noted by Max et al. [58]. This suggests the broader applicability of tDCS in terms of addressing various facets of BED beyond specific behaviors, highlighting its potential as a comprehensive treatment approach. 

## 7. Mechanisms of Action of tDCS in Binge Eating Disorder

Defined, if somewhat overlapping, brain areas and neurocircuitry control food-related appetite, decision making, executive function, and impulsivity. For instance, the triggering of food hunger involves a vast brain network that includes the amygdala [65], lateral hypothalamus [66], ventral striatum (nucleus accumbens) [67], and ventral tegmental region [68]. On the other hand, goal-directed and habitual decision making is primarily coordinated by the dorsal striatum, which is further subdivided into the dorsomedial striatum (caudate) and the dorsolateral striatum (putamen) [69]. The primary neurological basis of executive function is the prefrontal cortex, particularly the lateral prefrontal cortex [70]. Although the precise anatomical correlates of impulsivity remain unknown, it is thought that several brain areas, including the striatum, temporal pole, insula, anterior cingulate cortex, prefrontal cortex, and hippocampus, are implicated [69]. Due to its widely distributed receptors in the brain regions and neurocircuitry, implicated in food craving [71], decision making [72], executive function [73], and impulsivity [74], as well as its functional associations with these risk factors, the neurotransmitter dopamine has garnered increasing attention in the field of binge eating.

Mesolimbic circuits are formed by dopamine neurons in the ventral tegmental area [75] sending projections to the ventral striatum [69], the primary brain region related to food cravings [69]. Motivation has long been linked to the mesolimbic dopaminergic system [76]. The commencement of food consumption is facilitated by the hyperactive mesolimbic dopaminergic system, which in the context of eating habits increases incentive salience or cravings for food-related rewards [77,78].

The evidence from most studies indicates that people with BED have reduced dopamine levels and reduced dopamine activity [69]. The concept that a hypodopaminergic condition causes binge eating is in line with reward-related models or theories that address drug addiction (dopamine desensitization theory) [79], obesity (dynamic vulnerability model) [80], and alcohol-use disorder (three-stage model) [81]. The reward surfeit and reward deficit hypotheses are two competing dopamine-related ideas that are discussed in the literature on obesity [69]. The former suggests that eating high-energy foods enhances reward responsiveness, or dopamine signaling, which in turn raises the risk of obesity; the latter suggests that the opposite occurs [69].

It has been shown that applying tDCS to the frontal areas causes the release of dopamine into the striatum [82,83]. This proves the effects of tDCS on the dopaminergic system and the reward system. Since BED is highly correlated with substance-use disorder [84], and because both conditions share common neurobiological underpinnings related to interruptions in the dopaminergic pathways, tDCS may offer a potential avenue for intervention. Considering the dopamine desensitization theory of drug addiction [79], tDCS could be explored as a tool to modulate dopamine release during different stages of BED. By targeting the striatum, which is associated with reward processing, tDCS may influence dopamine release and help to regulate the reward-related mechanisms implicated in both binge eating and substance use and may potentially counteract the decreased dopamine release and downregulated responses associated with chronic binge eating. Similarly, in alignment with the three-stage model of alcohol dependence, tDCS might be applied to target the ventral striatum during the binge/intoxication stage. This could potentially mitigate the positive reinforcement processes that contribute to the initial transition into addiction. Additionally, tDCS might be explored in addressing compromised dopamine functions during the withdrawal/negative affect stage, potentially reducing sensitivity to rewards and tolerance.

The prefrontal cortex is the primary regulator of inhibitory control over behavior [85]. Dysregulations and lower baseline activity in PFC areas have been identified in persons with BED [86]. In addition, there are deficiencies in inhibitory control [87], especially in the medial prefrontal cortex (mPFC) [87], and we see increased activation in response to food signals [88]. Two competing systems are conceptualized: a “STOP” system that is damaged in compulsive eating and a “GO” system that is sensitized in obsessive eating [89]. The “GO” system’s brain activity may have been modulated by tDCS when applied to the right DLPFC. Through focusing on the hyper-responsive regions linked to food cues, tDCS may have regulated or diminished the heightened reactivity to stimuli that cause cravings in people with BED. In the case of compulsive eating, this modulation may have improved inhibitory control and decreased impulsivity by creating a better balance between the “GO” and “STOP” systems.

## 8. Limitations and Prospects for Further Research

Although the results from the studies included in this review are promising, there are several factors that should be considered in the design of future studies of tDCS in BED. 

### 8.1. Heterogeneity in Methodologies

There is significant methodological variation across the included research, which makes it difficult to combine data and reach firm conclusions. Methodological diversity is influenced, for example, by variations in the tDCS application with respect to reference electrode montage. Furthermore, the lack of a consistent framework for outcome measurements contributes to methodological variability, with some studies emphasizing psychopathological aspects or inhibitory control while focusing mostly on self-reported bouts of binge eating. 

### 8.2. Lack of Consistency in Outcome Measures

The studies included in this review demonstrate significant inconsistency in the choice of outcome measures, making it more difficult to develop a coherent picture of how tDCS affects BED. The lack of consistent evaluations amongst studies makes it more difficult to synthesize the data and restricts how broadly the conclusions can be applied. While some studies use measures of inhibitory control or psychopathological characteristics linked to BED, others concentrate mostly on self-reported BED episodes. For example, heterogeneity is introduced into the assessment of the frequency, food intake, food cravings, and desire to binge eat when a variety of tools are used, such as the Binge Eating Scale (BES), Eating Disorder Examination (EDE), Dutch Eating Behavior Questionnaire-Restraint Subscale (DEBQ-R), and Palatable Eating Motives Scale (PEMS). Future studies should place a high priority on adopting standardized outcome measures in order to resolve this discrepancy and guarantee a thorough and coherent assessment of the effects of tDCS on BED. The development of a consensus regarding the essential evaluation instruments associated with binge eating behavior, inhibitory control, and psychopathological characteristics would improve the validity and applicability of results. Furthermore, establishing a common set of outcome measures for tDCS trials in BED will help with meta-analyses and lead to a more sophisticated comprehension of the intervention’s effects. 

### 8.3. Limited Sample Sizes and Diversity

The inclusion of very small sample sizes, which may jeopardize the findings’ robustness and generalizability, as a common issue seen in the examined studies. Some studies had small sample sizes, casting doubt on their statistical power and capacity to identify meaningful effects. The results’ external validity was further limited by the variation in participant characteristics, such as age, gender, and body mass index (BMI). Several studies were primarily focused on demographic groups, such as samples that were primarily female or those within a particular BMI range. This limited the applicability of the findings to a larger community of people with BED. The lack of varied participant profiles restricted our ability to comprehend the potential effects of tDCS on various BED population subgroups. Future studies should put an emphasis on using larger and more varied sample sizes to solve these constraints. Using more participants with a greater range of ages, different gender identities, and varied BMI categories will improve the study’s external validity and enable a more thorough investigation of the effects of tDCS on various subpopulations. A more nuanced assessment of the intervention’s effectiveness and its potential relevance to a variety of demographic groups impacted by BED will be made possible by using robust and inclusive sampling methodologies. 

### 8.4. Lack of Long-Term Follow-Up

The majority of the examined studies’ have the conspicuous disadvantage of reliance on comparatively brief follow-up evaluations, which raises questions regarding the long-term sustainability and durability of the benefits of tDCS on BED. Numerous studies that were part of this evaluation evaluated results from just after the intervention, providing little information about how long-lasting the apparent gains will be. Our comprehension of the long-term effects of tDCS on binge eating behavior, inhibitory control, and psychopathological traits linked to BED is hampered by the lack of strong long-term follow-up data. Since BED is frequently accompanied by chronicity, assessing tDCS’s potential as a long-term therapeutic intervention requires an understanding of its long-term impacts. Future studies should include thorough long-term follow-up evaluations to track the trajectory of the effects of tDCS over a prolonged period in order to address this shortcoming. This will offer insightful information about sustaining treatment benefits, preventing relapses, and maintaining the general stability of the noted improvements. Including extensive follow-up periods in longitudinal studies will enhance our comprehension of the temporal dynamics of the effects of tDCS in BED patients.

### 8.5. Potential Sex-Specific Effects

tDCS may have sex-specific impacts on the outcomes of BED according to a significant finding from the evaluated research. While most studies offer insightful information on the general effectiveness of tDCS, there is growing evidence that suggests gender may have a role in how tDCS affects food cravings and the urge to binge eat. Research by Burgess et al. [54] and Ray et al. [63] demonstrates that men and women respond differently to tDCS, with men exhibiting a more marked decrease in food cravings and the urge to binge eat. This suggests that the effects of tDCS may be modulated differently depending on a person’s sex, highlighting the need for more research and greater comprehension of gender-related variations in treatment outcomes. Future studies should use gender-specific stratified analyses to address this possible sex specificity and separate the complex effects of tDCS in BED patients who are male and female. Investigations should also look into any underlying causes of these sex-specific variances, such as hormone changes or psychological issues. The refinement of treatment techniques and customization of therapies based on individual characteristics will come from a more thorough study of the interaction between tDCS, sex, and BED outcomes. 

### 8.6. Influence of Expectations

The impact of participant expectations on the results of tDCS in BED is another feature highlighted by the evaluated studies. Several studies, such as the work of Ray et al. [64], indicate that participant expectations may have a major influence on how well tDCS reduces appetite and eating habits. Regardless of the actual type of stimulation, Ray et al. [64] showed that people who thought they were receiving active tDCS performed better in terms of lowering appetite and eating than people who thought they were in the sham tDCS group. This emphasizes how crucial it is to take psychological aspects like participant expectations into account when analyzing the results of tDCS. Future studies should include techniques to rigorously regulate and evaluate participant expectations to address the impact of expectations. This could entail applying placebo-controlled designs, evaluating participant perceptions of the kind of stimulation they received, and investigating the relationship between expectations and tDCS results. Comprehending the influence of expectations on results will lead to a more sophisticated analysis of tDCS effectiveness in BED and help design individualized treatment plans that consider psychological aspects.

### 8.7. Influence of Genetics

The effect of tDCS on appetite may depend on genetics, as shown by a study by Fassini et al. [90]. In this study, DLPFC stimulation was performed in 38 obese women. participants were categorized based on the existence or lack of a Met allele (associated with decreased COMT enzyme activity). The findings demonstrated that whereas the acitivity of Met carriers tDCS saw the greatest drop in appetite over time, non-Met carriers receiving active tDCS maintained high levels of apetite. Therefore, future studies should perform genetic testing for the presence of the Met allele to check whether the reduction in appetite in BED patients depends on the participants’ genetic variant. When selecting appropriate genetic polymorphisms associated with BED, we recommend using the review by Manfredi et al. [91].

### 8.8. Considering Comorbidities as Confounding Factors

As stated in the introduction, BED is often accompanied by comorbidities that may complicate treatment outcomes. Future studies on tDCS in BED should use detailed psychiatric diagnostics of patients to determine their psychiatric condition and include or exclude patients with a given disease to control for its impact on treatment outcomes.

### 8.9. Incorporation of Neurophysiological and Neuroimaging Measurements

Little is known about the mechanisms of action of tDCS in BED. In addition to the behavioral measurements used so far, future studies need to use new measurement methods, such as electroencephalography (EEG), functional magnetic resonance imaging (fMRI), and positron emission tomography (PET).

### 8.10. Methodological Limitations of This Review

In the methods section, we utilized approaches that are typically associated with systematic reviews to enhance the rigor and quality of our study. However, it is important to note that our review does not strictly adhere to the criteria outlined by the PRISMA guidelines for systematic reviews and meta-analyses. While our methodology draws on elements commonly found in systematic reviews, our study is distinct in its scope and methodology.

## 9. Conclusions

There is growing interest in the use of tDCS in eating disorders, including binge eating disorder. The evidence to date supports the effectiveness of stimulation in improving symptoms of BED, such as food craving, food intake, number of binge eating episodes, and inhibitory control. However, conclusions should be treated with caution. There is little research on tDCS in BED, and the total sample of patients is also small. Further RCTs are needed, with sufficiently large and homogeneous patient samples, to establish the use of this treatment.

## Figures and Tables

**Table 1:** Summary of main findings from articles included in the review.

Author, Citation	Population	Used Test	Interventions	Stimulation Site	Current Intensity	Duration (min)	Main Findings in Treatment Group
Max et al. [53]	31 participants: 1 mA condition (n = 16), 2 mA condition (n = 15)	Self-reported frequency of binge eating episodes, antisaccades task	1 session of real or sham tDCS	Anode on right DLPFC (F4), cathode on the left deltoid muscle	1 mA or 2 mA	20	- A significant decrease in self-reported binge eating episodes over time was observed only in the group that had 2 mA stimulation, and there was no change in the group that had 1 mA stimulation. - All patients improved over the three measurement points concerning error rate and the latencies of correct antisaccades. The stimulation did not affect error rates, but the group that had 2 mA stimulation improved with faster latencies of correct antisaccades compared to sham stimulation, and the group that went through 1 mA stimulation showed slower latencies.
Burgess et al. [54]	30 participants	Binge Eating Scale (BES), Dutch Eating Behavior Questionnaire-Restraint Subscale (DEBQ-R), Palatable Eating Motives Scale (PEMS)	1 session of real or sham tDCS	Anode on the right DLPFC (F4), cathode on the left DLPFC (F3)	2 mA	20 min	- There was no effect of tDCS on binge eating frequency. - tDCS significantly reduced food intake, as measured by DEBQ-R. Participants ate fewer total kcals in the lab after tDCS (614.50 ± 55.5) versus after sham (689.54 ± 60.8) treatment, with an 11% reduction. After tDCS treatment, participants ate 324.72 ± 30.5 kcals of the preferred food versus the 393.52 ± 36.3 kcals eaten after sham treatment, and there was a 17.5% reduction. - tDCS significantly reduced food craving, as measured by PEMS. The decreased intake of palatable food for Reward Enhancement motives accounted for 20% of the variance in terms of reducing food craving. Additionally, tDCS significantly reduced the desire to binge eat, which was also measured by PEMS. An r-ANOVA showed that tDCS reduced desire to binge eat on the day of the stimulation, roughly 5–6 h post-stimulation, in men only.
Gordon et al. [55]	15 participants: real tDCS (n = 6), sham tDCS (n = 9)	Not reported, frequency of binge eating	6 sessions	Anode on the right DLPFC (F4), cathode on the left DLPFC (F3)	2 mA	Not reported	- A reduction in binge eating episodes was observed by six participants (ABM and real tDCS n = 2; ABM and sham tDCS n = 4), ranging from ‘a little bit’ to a ‘huge difference’.
Giel et al. [56]	41 participants	Eating Disorder Examination (EDE), used to assess inhibitory control capacity, measured the course of the error rate (%) of the food-related eye-tracking task from the beginning of the training (T1) over each training session until immediately after the training (T7) compared to baseline (T0) values and the results of the Three-Factor Eating Questionnaire (TFEQ).	6 sessions	Anode on the right DLPFC (F4), no reference electrode data	2 mA	20 min	- In the real tDCS group, the BE frequency was reduced from 18.6 to 4.4 (T8) and to 3.8 (T9). - The results showed significant improvement in inhibitory control over the training period and showed a significant effect at T7 as compared to T0 in both study arms. - The results in TFEQ showed that this measure decreased from 10.6 (T0) to 9.3 (T7 and T8) after real tDCS.
Beaumont et al. [57]	17 participants	Appetite visual analogue scale (VAS), the Food Craving Questionnaire-State (FCQ-S), the Leeds Food Preference Questionnaire (LFPQ), Control of Eating Questionnaire (CoEQ)	2 sessions, real or sham tDCS	Anode on the right DLPFC (F4), cathode on the occipital zero point (Oz)	2 mA	20 min	- VAS hunger levels following active tDCS treatment grew to meet those of post-sham stimulation, indicating a substantial change from pre- to post-tDCS (F(1, 15) = 6.796, *p* = 0.020, = 0.312, BF10 = 0.188). This effect was no longer significant (F(1, 30) = 0.610, *p* = 0.441, = 0.020, BF10 = 0.680) when baseline hunger was taken into account. - When comparing measures of fullness (F(1, 15) = 1.282, *p* = 0.275, = 0.079, BF10 = 0.040), prospective consumption (F(1, 15) = 2.606, *p* = 0.127, = 0.148, BF10 = 0.063), and desire to eat (F(1, 15) = 1.452, *p* = 0.247, = 0.088, BF10 = 0.054), there were no significant differences observed between the active and sham tDCS. Bayes factors suggested moderate-to-strong evidence in favor of the null hypothesis. - Regarding LFPQ, all assessments showed no significant impacts, with the exception of explicit desire and preference for HFSW foods. The preference for HFSW foods rose after active tDCS and fell after sham tDCS for both explicit like and desire. ANCOVA was used to assess if the difference in baseline hunger was the cause of these substantial effects; when hunger was taken into account, the difference between pre- and post-tDCS phases ceased to be significant. - Regarding FCQ-S, scores for food cravings after active versus sham procedures did not differ. In the CoEQ assessment, results for savory food cravings were close to significant (active 45.3 ± 17.9 mm, sham 49.4 ± 20.6 mm) (t(13) = 2.128, *p* = 0.053), indicating that savory food cravings decrease when active procedures are followed.
Max et al. [58]	31 participants: real tDCS (n = 15), sham tDCS (n = 16)	Food Craving Questionnaire-State (FCQ-S), Eating Disorder Examination (EDE)	6 sessions	Anode on the right DLPFC (F4), cathode was positioned extracephalic on the left deltoid muscle	2 mA	20	- The number of binge eating episodes decreased (from 17.35 to 6.26). - The total FCQ score improved (from 38.26 to 37.97). - The EDE score improved (from 2.61 to 2.04).
